# Impaired Glomerular Filtration Rate in Type 2 Diabetes Mellitus Subjects: A Nationwide Cross-Sectional Study in Thailand

**DOI:** 10.1155/2020/6353949

**Published:** 2020-08-12

**Authors:** Naowanit Nata, Ram Rangsin, Ouppatham Supasyndh, Bancha Satirapoj

**Affiliations:** ^1^Division of Nephrology, Department of Medicine, Phramongkutklao Hospital and College of Medicine, Bangkok, Thailand; ^2^Department of Military and Community Medicine, Phramongkutklao College of Medicine, Bangkok, Thailand

## Abstract

**Background:**

Type 2 diabetic mellitus (T2DM) patients with impaired renal function have a higher risk of mortality, and often progress to end-stage renal disease. The study aims to determine the prevalence of kidney disease and investigate the relationship between various factors and impaired renal function in a large population of patients with T2DM.

**Methods:**

We conducted a cross-sectional study among 30,377 patients from a nationwide diabetes study involving 602 Thai hospitals. Impaired glomerular filtration rate (GFR) was defined as <60 mL/min per 1.73 m^2^. Multivariate logistic regression was used to determine the association between standard risk factors and impaired GFR.

**Results:**

The prevalence of impaired GFR in a T2DM population was 39.2%. After adjusting for multiple risk factors, advanced age (adjusted OR 11.69 (95%CI = 3.13 to 43.61)), macroalbuminuria (adjusted OR 3.54 (95%CI = 1.50 to 8.40)), high serum uric acid (adjusted OR 2.06 (95%CI = 1.73 to 2.46)), systolic BP 130-139 mmHg (adjusted OR 3.21 (95%CI = 1.30 to 7.96)), hemoglobinA1C (HA1C) <6% (adjusted OR 3.71 (95%CI = 1.65 to 8.32)), and HA1C >7% (adjusted OR 2.53 (95%CI = 1.38 to 4.63)) were found to be associated with a significantly increased risk of impaired GFR among T2DM patients.

**Conclusion:**

Almost 40% of patients with T2DM in a nationwide cross-sectional study in Thailand had impaired GFR. Advanced age, albuminuria, hyperuricemia, hypertension, HA1C <6%, and HA1C >7% were independently associated with increased prevalence of impaired GFR.

## 1. Background

In 2010, the worldwide adult population with type 2 diabetes mellitus (T2DM) was estimated to be about 285 million and by 2030 an increase of 54% to about 439 million is predicted, reflecting a remarkable increase in renal complications [1]. Patients with T2DM with impaired renal function have become an important public health problem [2], and approximately 40% of patients with diabetes develop kidney disease resulting in albuminuria reduced glomerular filtration rate (GFR) or both [3]. The presence of impaired GFR and high levels of renal injury biomarkers had a more rapid decline in renal function and progression of renal disease [4, 5]. Moreover, patients with T2DM with renal impairment and/or albuminuria have an increased mortality risk, especially a higher risk of cardiovascular death, when compared with other diabetic patients without renal impairment [6].

Prevalence rates of chronic kidney disease (CKD) in the general population and among patients with T2DM in Thailand have been documented at a significantly higher rate than those previously reported in other populations [7–9]. Significantly impaired renal function is defined as estimated GFR less than 60 mL/min/1.73 m^2^, while markers of kidney damage include albuminuria and/or proteinuria. The Kidney Disease: Improving Global Outcomes (KDIGO) Guidelines recommend staging of CKD based on the underlying cause, estimated GFR, and level of albuminuria. T2DM with CKD often progresses to end-stage renal disease (ESRD). Thus, risk factors that are identified and treated at an early stage may prevent or slow the progression to ESRD in T2DM. Several studies have been conducted on the prevalence of CKD and forms of renal disease among patients with T2DM in Asian countries [10–13]. Limited studies have been conducted in a randomly selected T2DM population sample in Thailand [8, 9]. Moreover, the lack of general hospitalized-based screening programs in Thailand has led to patients with T2DM being detected with advanced CKD stage. Quite possibly, early detection of risk factors of kidney disease might have an impact on this problem through earlier intervention. The authors conducted a cross-sectional study to determine the prevalence of kidney disease among patients with T2DM and investigated the relationship between various factors and impaired renal function in a large population of patients with T2DM in a nationwide survey in Thailand.

## 2. Methods

### 2.1. Study Population

The present study comprised a nationwide, multicenter, cross-sectional survey of outpatients with T2DM across Thailand. The analysis was performed using the Diabetes Dataset of Medical Records, an ongoing nationwide project, collected between 2013 and 2014. This project was administered by the Medical Research Network of the Consortium of Thai Medical Schools (MedResNet) Thailand, under the sponsorship of the Thai National Health Security Office. Patients and hospitals were sampled using a proportional to size stratified cluster sampling approach, and then, outpatients with T2DM were proportionally sampled from the 602 participating hospitals across Thailand. The study was approved by the Ethics Review Committee for Research in Human Subjects, Ministry of Public Health, Thailand. Informed consent was obtained from all patients.

Patients with T2DM, receiving medical care in participating hospitals for at least 12 months, were included in the present study. T2DM was reviewed using retrieved medical and personal data, including baseline demographic characteristics, hypertension, use of antihypertensive or lipid-lowering medications, comorbidities, available electrocardiogram (ECG) data and results, and biochemical variables including urine albumin, serum creatinine, uric acid, total cholesterol, HDL-cholesterol, LDL-cholesterol, triglycerides, fasting plasma glucose levels, and hemoglobinA1C (HA1C). Only patients with serum creatinine available were included in the study. Exclusion criteria included acute kidney injury episode, pregnancy, unspecified type of DM, and patient life expectancy <1 year.

Trained research nurses and technicians administered a structured questionnaire and performed blood pressure (BP) and anthropometric measurements. BP was measured using a mercury sphygmomanometer and included three measurements. The mean of the second and third systolic and diastolic BP measurements was used in the analysis. Body weight, height, waist circumference, and hip circumference were measured according to standard protocol. Body mass index (BMI) was calculated as weight in kilograms divided by height in meters squared. All participants received treatment based on the standard strategies for diabetes, hypertension, and hyperlipidemia.

The presence of impaired GFR or CKD was assessed by measuring serum creatinine. An estimate of the GFR was obtained by the Chronic Kidney Disease Epidemiology Collaboration (CKD-EPI) equation [14]. Patients were assigned to one of the following categories of eGFR: 1 (≥90), 2 (60–89), 3 (30–59), 4 [15–29], and 5 (<15 mL/min/1.73 m^2^). Finally, subjects were classified as having no CKD or CKD on the basis of the value of GFR <60 mL/min per 1.73 m^2^, according to the National Kidney Foundation's Kidney Disease Outcomes Quality Initiative.

### 2.2. Statistical Analysis

Continuous data were described as mean and standard deviation (SD). Categorical variables were described in percentage. Unpaired Student's *t*-test was used to compare continuous variables and the chi-square test was used to evaluate proportions between groups. To determine associations with impaired GFR and various factors, we first examined the unadjusted relationships (odds ratio (OR) with 95% confidence intervals (CI) and then adjusted the models for age, sex, BMI, waist circumference, smoking, systolic BP, diastolic BP, HA1C, hemoglobin, serum uric acid, triglycerides, HDL-cholesterol, and albuminuria using multivariate logistic regression analysis. All results were considered significant when *P* value was <0.05.

## 3. Results

**Figure 1:**
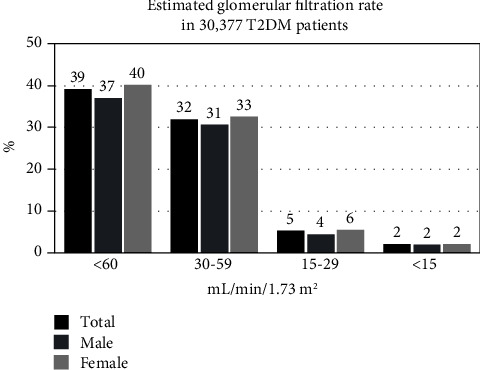
Prevalence of kidney disease according to GFR in a T2DM population.

A total of 30,377 patients were included in the study of whom 11,909 (39.2%) had GFR <60 mL/min per 1.73 m^2^. The majority with impaired GFR were at stage 3 (*n* = 9,729, 32%) with a few patients at stage 4 CKD (*n* = 1,566, 5.2%) or stage 5 CKD (*n* = 614, 2%) (Figure 1). One single random UACR was obtained among 3,243 patients (10.6%). The average duration of diabetes was 6.9 ± 4.7 years. The mean HbA1C was 7.98 ± 2.07%, and 31.3% of patients had an HbA1C <7%. The mean systolic and diastolic BP were 131.7 ± 13.3 and 75.0 ± 8.1 mmHg, respectively, while 44.9% of patients had a systolic BP <130 mmHg and 70.7% of patients had a diastolic BP <80 mmHg.

The clinical characteristics of patients with and without impaired GFR are shown in Table 1. In the entire population, 76.8% had hypertension, 69.9% had dyslipidemia, 7.1% had diabetic neuropathy, 6.6% had coronary heart disease, 4% had gout, 3.1% had cerebrovascular disease, and 0.6% had peripheral arterial disease as comorbid diseases. All comorbid diseases except dyslipidemia were significantly higher among patients with T2DM with impaired GFR. Age, percentage of females, and systolic BP were higher (*P* < 0.001) among patients with impaired GFR. Percentage of factors related to smoking, body weight, BMI, diastolic BP, and waist circumference was lower (*P* < 0.001) among patients with impaired GFR (Table 1).

The current medications of patients with and without impaired GFR are shown in Table 2. For BP-lowering agents, the percentage of those using diuretics, antiadrenergic drugs, calcium channel blockers, and vasodilators and females was higher (*P* < 0.001) among patients with impaired GFR. For glycemic and lipid-lowering agents, the percentage of those using biguanides, sulfonylurea, and thiazolidinedione was lower (*P* < 0.001) among patients with impaired GFR, but the percentage of those using insulin and fibrates was higher (*P* < 0.001) among patients with impaired GFR.

Table 3 displays the patient data according to impaired GFR status. The mean values of estimated GFR among patients with and without impaired GFR were 41.8 ± 13.3 mL/min/1.73 m^2^ and 85.4 ± 16.8 mL/min/1.73 m^2^, respectively. The impaired GFR patient group had significantly lower fasting plasma glucose, HA1C, hemoglobin, and HDL-cholesterol and had higher serum uric acid, serum potassium, total cholesterol, triglycerides, and albuminuria.

To identify putative risk factors associated with impaired GFR, we performed multivariate logistic regression analysis in the model for all variables. A significant association was found regarding various clinical and laboratory factors with impaired GFR in the entire population as an unadjusted risk ratio as shown in Table 4. After adjusting for multiple factors, advanced age, high systolic BP, HA1C <6%, HA1C >7%, high serum uric acid, and high albuminuria were associated with a significantly increased risk of impaired GFR. Diastolic BP differed significantly among individuals with and without impairment but was not independently associated with impaired GFR.

Compared with age <50 years, the multivariate-adjust odds for impaired GFR of 60-69 years, and >70 years were 11.69 (95%CI = 3.13 to 43.61) and 21.85 (95%CI = 5.64 to 84.69), respectively. The multivariate-adjust odds for impaired GFR of systolic BP 120-129 mmHg and 130-139 mmHg were 2.72 (95%CI = 1.05 to 7.00) and 3.21 (95%CI = 1.30 to 7.96), respectively, compared with systolic BP <120 mmHg. The multivariate-adjust odds for impaired GFR of albuminuria 30-300 mg/gCr and > 300 mg/gCr were 2.47 (95%CI = 1.39 to 4.40) and 3.54 (95%CI = 1.50 to 8.40), respectively, compared with albuminuria <30 mg/gCr (Table 4).

**Figure 2:**
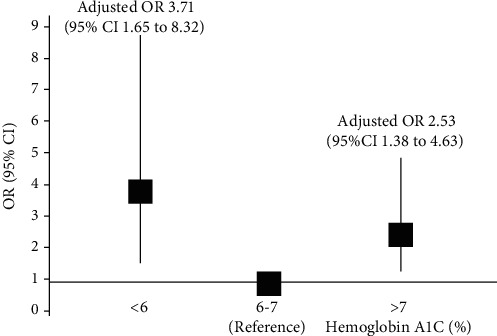
Adjusted OR between hemoglobin A1C (HA1C) and impaired GFR among patients with T2DM. Adjusted by age, sex, BMI, waist circumference, smoking, systolic BP, diastolic BP, HA1C, hemoglobin, serum uric acid, triglycerides, HDL-cholesterol, and albuminuria.

The multivariate-adjusted odds for impaired GFR of HA1C <6%, and >7% were 3.71 (95%CI = 1.65 to 8.32) and 2.53 (95%CI = 1.38 to 4.63), respectively, compared with HA1C 6-7% (Table 4). Finally, a clear J-shaped relationship was observed between HA1C levels and impaired GFR in adjusted models (Figure 2).

Univariate analysis showed a significant association of impaired GFR with female, aging, low BMI, smoking, high systolic BP, low diastolic BP, low HA1C, low hemoglobin, high serum uric acid, hypercholesterolemia, hypertriglyceridemia, low HDL, and high albuminuria (Table 5). Multiple logistic regression analysis after adjusting potential factors revealed aging [adjusted OR 95% CI 1.09 (1.07 to 1.12)], systolic BP [adjusted OR 95% CI 1.02 (1.00 to 1.04)], HA1C [adjusted OR 95% CI 1.16 (1.04 to 1.31], hemoglobin [adjusted OR 95% CI 0.85 (0.74 to 0.98)], serum uric acid [adjusted OR 95% CI 1.90 (1.64 to 2.20)], and albuminuria [adjusted OR 95% CI 1.01 (1.01 to 1.02)] were still significantly associated with impaired GFR in patients with T2DM (Table 5).

## 4. Discussion

To the best of our knowledge, this comprised a large nationwide study to assess the prevalence of kidney disease and associated factors among patients with T2DM in Thailand. In this study, we found that almost 40% of 30,377 studied patients with T2DM had GFR less than 60 mL/min/1.73 m^2^. Advanced age, albuminuria, high serum uric acid, high systolic BP, HA1C <6%, and HA1C >7% were the major factors associated with impaired GFR in multivariable models.

Data from the United Kingdom Prospective Diabetes Study (UKPDS) demonstrated that after 15 years, approximately 28% of patients had significant albuminuria [3], and another study from the UK showed a prevalence of clinically and significantly impaired GFR less than 60 mL/min/1.73 m^2^ of 31% among patients with T2DM [15]. In addition, data from Asian populations also showed the prevalence of albuminuria and CKD among patients with T2DM was approximately 25-40% and 30%, respectively [10–13]. Therefore, the prevalence of impaired GFR reported in our study was similar to that seen in previous reports.

Microvascular complications among patients with T2DM are a consequence of prolonged hyperglycemia. Many studies have reported the impact of poor glycemic control and albuminuria on the development of diabetic complications [16–18]. Similarly, a prospective study of patients with T2DM confirmed that baseline albuminuria and poor glycemic control were important initiators as well as accelerators for the progression of albuminuria and development of diabetic nephropathy [19, 20]. Our findings confirmed that the optimal glycemic control with HA1C at 6-7% revealed a significantly low risk of impaired GFR. However, our study indicated that low HA1C, <6%, was associated with impaired renal function. Probably, this was because glucose homeostasis is extremely altered among patients with T2DM with CKD, who are exposed to a high risk of both hyperglycemia and hypoglycemia [21]. Glycemic control in advanced CKD improves spontaneously with the progression of declining GFR especially at time of initiating dialysis therapy, leading to low hemoglobinA1c levels (<6%), and some patients required cessation of hypoglycemic agents and insulin [22]. The main reason for improving glycemic control in advanced CKD is postulated to be impaired renal insulin degradation and clearance, reduced renal gluconeogenesis, and uremic malnutrition [23]. Moreover, these factors can contribute to a lower than usual threshold for clinical hypoglycemia, which is a common complication among patients with advanced CKD and undergoing dialysis [24].

Our study documented a strong positive correlation between advanced age and increased risk of decreased GFR among patients with T2DM. The findings in the present study are consistent with related studies, indicating age was an independent risk factor in the development of CKD in the general population [25] and among individuals with T2DM [19, 20]. Among hypertensive patients with CKD, BP control is essential to minimize the progression of CKD and reduce CKD-related complications. The most common predictor of the progression to diabetic nephropathy and albuminuria among patients with T2DM is uncontrolled systolic BP [12]. Our findings confirmed that optimal systolic BP control, <120 mmHg, was associated with significantly low risk of impaired GFR similar to previous findings. However, in our study, diastolic BP control was lower among patients with decreased GFR, and high levels of diastolic BP were not independently related to impaired GFR. Our results are also similar to those reported in the Reduction in Endpoints in NIDDM in the Angiotensin II Antagonist Losartan (RENAAL) study. The RENAAL study showed that increases in systolic BP increased the risk for ESRD, but this relationship was not seen for diastolic BP [26]. In addition, 70% of diastolic BP control obtained in our population was much higher than those published in a related study [27], so this improvement should be interpreted with caution, because individuals with no BP data over the study period were not included in our analysis.

Hyperuricemia is highly prevalent among patients with CKD, and it independently predicted the development of T2DM [28]. Hyperuricemia was also associated with an increased risk of CKD and ESRD in cross-sectional and long-term cohort studies [29–31]. The results of the present study also suggested that in the Thai T2DM population, high serum uric acid levels are correlated with increased prevalence of impaired GFR. Experimental studies have confirmed that uric acid can accelerate renal injury in animal models via a mechanism linked to increased blood pressure, COX-2-mediated vascular injury, tubulointerstitial fibrosis, and cell infiltration as well as arteriolopathy of the preglomerular vessels [32, 33]. Thus, these studies, including our study, raised the possibility that high uric acid levels may mediate renal disease and progression.

The duration of diabetes is a very important factor in the development of diabetic kidney disease. Several studies have indicated that the duration of diabetes was related to the severity of nephropathy, especially when it first appears 10-15 years after the onset of T1DM and after 5-10 years among patients with T2DM [34, 35]. In contrast, the average duration of diabetes was 6.9 years with 40% of impaired GFR in our study. However, differences in race and in various other risk factors for developing CKD in T2DM probably have an important role as well. The majority of patients in our study were at high risk for CKD progression including advanced age, poor glycemic control (70% of patients had an HbA1C ≤7%), and uncontrolled hypertension (55% of patients had a systolic BP >130 mmHg).

The prevalence of coronary heart disease was relatively low in our study, because coronary heart disease was defined as myocardial infarction or history of coronary revascularization in only medical records and data on ECG testing was available only 6,531 patients (21.5%) in the study. Moreover, the prevalence of coronary heart disease was 1% in the Thai general population [36]. In contrast, Caucasian patients with T2DM at age 45-75 years showed a higher prevalence of coronary heart disease [37]. However, our study included patients with T2DM aged from 35 to 75 years.

This study has several strengths. The data were carefully collected. The study population included a large sample of patients with T2DM in an Asian population and all serum creatinine assays were made in a standard laboratory using the enzymatic method assay. However, the study had several limitations. First, data on albuminuria was unavailable among all patients with T2DM. Therefore, our results indicated such effects only for advanced CKD with GFR less than 60 mL/min/1.73 m^2^. Second, only a single serum creatinine value and single UACR was used to assess CKD. Therefore, distinguishing between patients with transient impairment of GFR and albuminuria and those with a persistent alteration was not possible. Additionally, serum creatinine was not measured in a single centralized laboratory, and this may have led to some variability in GFR estimation. Finally, because this was a hospital-based study, it could have introduced a referral bias and generalizability of results; therefore, it might be limited.

## 5. Conclusion

The patients with T2DM in the Thai national survey had significantly impaired GFR (40%). This suggested that CKD was a major concern for patients with T2DM in Thailand. Aging, systolic hypertension, albuminuria, poor glycemic control, and hyperuricemia were independently associated with increased prevalence of impaired GFR. Our data supports that the early detection of these factors should be a routine strategy to prevent CKD in Thailand.

## Figures and Tables

**Table 1:** Characteristics of the patients with T2DM by impaired GFR status.

Baseline profiles	Total *N* = 30,377	Impaired GFR <60 mL/min/1.73 m^2^ *N* = 11,909	Nonimpaired GFR ≥60 mL/min/1.73 m^2^ *N* = 18,468	*P* value
Age (years)	61.2 ± 10.9	66.4 ± 9.8	57.8 ± 10.3	<0.001
Female (n, %)	20955 (69%)	8424 (70.7%)	12531 (67.9%)	<0.001
Body weight (kg)	63.2 ± 12.8	61.1 ± 12.3	64.6 ± 12.9	<0.001
BMI (kg/m^2^)	25.5 ± 4.6	24.8 ± 4.4	25.9 ± 4.7	<0.001
Waist circumference (cm)	88.5 ± 10.6	87.9 ± 10.5	88.8 ± 10.7	<0.001
Smoking (n, %)	1346 (4.4%)	376 (3.2%)	970 (5.3%)	<0.001
Systolic BP (mmHg)	131.7 ± 13.4	133.6 ± 13.9	130.5 ± 12.8	<0.001
Diastolic BP (mmHg)	75.0 ± 8.1	73.7 ± 8.2	75.9 ± 7.9	<0.001
Comorbid diseases				
Hypertension (n, %)	23,342 (76.8%)	10,102 (84.8%)	13,240 (71.7%)	<0.001
Dyslipidemia (n, %)	21,238 (69.9%)	8,395 (70.5%)	12,843 (69.5%)	0.078
Gout (n, %)	1,204 (4%)	755 (6.3%)	449 (2.4%)	<0.001
Cerebrovascular disease (n, %)	946 (3.1%)	467 (3.9%)	479 (2.6%)	<0.001
Coronary heart disease (n, %)	2010 (6.6%)	1072 (9%)	938 (5.1%)	<0.001
Peripheral arterial disease (n, %)	181 (0.6%)	110 (0.9%)	71 (0.4%)	<0.001
Diabetic neuropathy (n, %)	2166 (7.1%)	1048 (8.8%)	1118 (6.1%)	<0.001

All values are expressed as mean ± SD and percentage.

Abbreviation: BMI: body mass index; BP: blood pressure; GFR: glomerular filtration rate.

**Table 2:** Blood pressure, glycemic and lipid lowering agents by impaired GFR status.

Medications	Total *N* = 30,377	Impaired GFR <60 mL/min/1.73 m^2^ *N* = 11,909	Non-impaired GFR ≥60 mL/min/1.73 m^2^ *N* = 18,468	*P* value
Blood pressure lowering agents				
Using any blood pressure lowering agents	22148 (72.9%)	9668 (81.2%)	12480 (67.6%)	<0.001
(i) Diuretics	6360 (20.9%)	3168 (26.6%)	3192 (17.3%)	<0.001
(ii) Antiadrenergic drugs	6139 (20.2%)	3007 (25.2%)	3132 (17%)	<0.001
(iii) Calcium channel blockers	11418 (37.6%)	5525 (46.4%)	5893 (31.9%)	<0.001
(iv) ACEI/ARB	16447 (54.1%)	6369 (53.5%)	10078 (54.6%)	0.063
(v) Vasodilators	1009 (3.3%)	739 (6.2%)	270 (1.5%)	<0.001
Glycemic lowering agents				
Using any glycemic lowering agents	29317 (96.5%)	11328 (95.1%)	17989 (97.4%)	<0.001
(i) Biguanides	21591 (71.1%)	6143 (51.6%)	15448 (83.6%)	<0.001
(ii) Sulfonylurea	19524 (64.3%)	6716 (56.4%)	12808 (69.4%)	<0.001
(iii) Thiazolidinedione	2527 (8.3%)	906 (7.6%)	1621 (8.8%)	<0.001
(iv) Alpha-glucosidase inhibitor	189 (0.6%)	65 (0.5%)	124 (0.7%)	0.174
(v) DPP-4 inhibitors	209 (0.7%)	89 (0.7%)	120 (0.6%)	0.315
(vi) GLP-1 agonists	18 (0.1%)	8 (0.1%)	10 (0.1%)	0.649
(vii) Insulin	6893 (22.7%)	3875 (32.5%)	3018 (16.3%)	<0.001
Lipid lowering agents				
Using any lipid lowering agents	21835 (71.9%)	8723 (73.2%)	13112 (71%)	<0.001
(i) Statin	18205 (59.9%)	7176 (60.3%)	11029 (59.7%)	0.351
(ii) Fibrate	4242 (14%)	1810 (15.2%)	2432 (13.2%)	<0.001

All values are expressed as percentage.

Abbreviation: ACEI: angiotensin converting enzyme inhibitor; ARB: angiotensin receptor blocker; DPP-4 inhibitors: dipeptidyl peptidase-4 inhibitors; GLP-1 agonist: glucagon-like peptide 1 receptor agonists.

**Table 3:** Laboratory profiles by impaired GFR status.

Laboratory profiles	Total *N* = 30,377	Impaired GFR <60 mL/min/1.73 m^2^ *N* = 11,909	Non-impaired GFR ≥60 mL/min/1.73 m^2^ *N* = 18,468	*P* value
BUN (mg/dL)	17.2 ± 10.5	22.9 ± 12.9	13.3 ± 5.8	<0.001
Serum creatinine (mg/dL)	1.2 ± 0.9	1.7 ± 1.3	0.8 ± 0.2	<0.001
Estimated GFR (mL/min/1.73 m^2^)	68.3 ± 26.3	41.8 ± 13.3	85.4 ± 16.8	<0.001
Fasting plasma glucose (mg/dL)	155.1 ± 49.9	152.9 ± 53.2	156.5 ± 47.6	<0.001
HemoglobinA1C (%)	7.9 ± 2.1	7.9 ± 2.1	8.0 ± 2.0	0.001
Hemoglobin (g/dL)	12.0 ± 2.5	11.3 ± 2.5	12.6 ± 2.3	<0.001
Serum potassium (mEq/L)	4.2 ± 0.8	4.3 ± 0.9	4.1 ± 0.5	<0.001
Serum uric acid (mg/dL)	5.9 ± 3.2	6.7 ± 2.4	5.5 ± 3.6	<0.001
Total cholesterol (mg/dL)	188.6 ± 46.6	190.5 ± 49.4	187.4 ± 44.8	<0.001
Triglycerides (mg/dL)	175.8 ± 111.9	186.9 ± 119.3	168.8 ± 106.4	<0.001
HDL-cholesterol (mg/dL)	47.2 ± 15.6	45.9 ± 16.5	47.9 ± 14.9	<0.001
LDL-cholesterol (mg/dL)	109.3 ± 38.0	109.6 ± 39.8	109.2 ± 36.8	0.399
Median UACR (mg/gCr)	30.1 (17, 118)	50 (30, 201.4)	30 (12.6, 82)	<0.001

All values are expressed as mean ± SD and percentage.

Abbreviation: BUN: blood urea nitrogen; GFR: glomerular filtration rate; HDL: high density lipoprotein; LDL: low density lipoprotein; UACR: urine albumin creatinine ratio.

**Table 4:** Multivariate logistic regression analysis between other factors on impaired GFR among patients with T2DM.

Factors	Crude OR (95% CI)	*P* value	Adjusted OR (95% CI)	*P* value
Female	1.15 (1.09, 1.2)	<0.001	1.01 (0.54, 1.91)	0.964
Age (years)				
<50	Reference	1	Reference	1
50-59	2.2 (2.00, 2.43)	<0.001	2.97 (0.77, 11.52)	0.114
60-69	5.39 (4.90, 5.93)	<0.001	11.68 (3.13, 43.61)	<0.001
>70	12.55 (11.36, 13.87)	<0.001	21.85 (5.64, 84.69)	<0.001
BMI (kg/m^2^)				
BMI ≤22.9	Reference	1	Reference	1
BMI 23-24.9	0.75 (0.70, 0.80)	<0.001	0.98 (0.46, 2.09)	0.950
Obese I, BMI 25-29.9	0.66 (0.63, 0.70)	<0.001	0.71 (0.33, 1.52)	0.375
Obese II, BMI ≥30	0.52 (0.48, 0.56)	<0.001	0.86 (0.29, 2.57)	0.780
Waist circumference (cm)	0.99 (0.99, 0.99)	<0.001	0.99 (0.95, 1.02)	0.423
Smoking	0.90 (0.84, 0.96)	0.002	1.35 (0.60, 3.01)	0.464
Systolic BP (mmHg)				
<120	Reference	1	Reference	1
120-129	1.15 (1.07, 1.24)	<0.001	2.72 (1.05, 7.00)	0.038
130-139	1.32 (1.23, 1.42)	<0.001	3.21 (1.30, 7.96)	0.012
>140	1.81 (1.68, 1.95)	<0.001	2.34 (0.92, 5.98)	0.075
Diastolic BP (mmHg)				
<80	Reference	1	Reference	1
80-89	0.64 (0.61, 0.68)	<0.001	0.52 (0.28, 1.03)	0.052
90-99	0.54 (0.47, 0.61)	<0.001	0.66 (0.15, 2.93)	0.581
≥100	0.94 (0.62, 1.45)	0.792	1.00 (0.00, 1.00)	0.999
HemoglobinA1C (%)				
<6	1.14 (1.05, 1.24)	0.002	3.71 (1.65, 8.32)	0.001
6-7	Reference	1	Reference	1
>7	0.87 (0.81, 0.92)	<0.001	2.53 (1.38, 4.63)	0.003
Hemoglobin (g/dL)	0.69 (0.68, 0.71)	<0.001	0.86 (0.73, 1.01)	0.071
Serum uric acid (mg/dL)	1.42 (1.38, 1.46)	<0.001	2.06 (1.73, 2.46)	<0.001
HDL-cholesterol (mg/dL)	0.99 (0.99, 0.99)	<0.001	1.00 (0.99, 1.02)	0.864
UACR (mg/gCr)				
<30	Reference	1	Reference	1
30-300	2.10 (1.77, 2.50)	<0.001	2.47 (1.39, 4.40)	0.002
>300	5.15 (3.77, 7.03)	<0.001	3.54 (1.50, 8.40)	0.004

All associations significant at *P*<0.05.

Abbreviation: BP: blood pressure; BMI: body mass index; UACR: urine albumin creatinine ratio.

^a^Adjusted by age, sex, BMI, waist circumference, smoking, systolic BP, diastolic BP, HA1C, hemoglobin, serum uric acid, triglycerides, HDL-cholesterol, and albuminuria.

**Table 5:** Univariate and multivariate logistic regression analysis to determine impaired GFR among patients with T2DM.

Factors	Univariate analysis		Multivariate logistic regression analysis	
	Odd ratio (95% CI)	*P* value	Odd ratio (95% CI)	*P* value
Female	1.15 (1.09, 1.20)	<0.001	0.86 (0.51, 1.45)	0.564
Age (years)	1.09 (1.08, 1.09)	<0.001	1.09 (1.07, 1.12)	<0.001
BMI (kg/m^2^)	0.95 (0.94, 0.95)	<0.001	0.98 (0.93, 1.02)	0.326
Smoking (yes)	0.90 (0.84, 0.96)	0.002	1.01 (0.51, 1.99)	0.987
Systolic BP (mmHg)	1.02 (1.02, 1.02)	<0.001	1.02 (1.00, 1.04)	0.030
Diastolic BP (mmHg)	0.97 (0.96, 0.97)	<0.001	0.98 (0.96, 1.01)	0.311
HemoglobinA1C (%)	0.98 (0.97, 0.99)	0.001	1.16 (1.04, 1.31)	0.009
Hemoglobin (g/dL)	0.69 (0.68, 0.71)	<0.001	0.85 (0.74, 0.98)	0.029
Serum uric acid (mg/dL)	1.42 (1.38, 1.46)	<0.001	1.90 (1.64, 2.20)	<0.001
Total cholesterol (mg/dL)	1.01 (1.01, 1.02)	<0.001	1.00 (0.99, 1.01)	0.966
Triglycerides (mg/dL)	1.01 (1.01, 1.02)	<0.001	1.01 (0.99, 1.02)	0.144
HDL-cholesterol (mg/dL)	0.99 (0.98, 0.99)	<0.001	1.00 (0.99, 1.01)	1.000
UACR (mg/gCr)	1.01 (1.01, 1.02)	<0.001	1.01 (1.01, 1.02)	0.045

Abbreviation: BP: blood pressure; BMI: body mass index; UACR: urine albumin creatinine ratio.

^a^Adjusted by age, sex, BMI, waist circumference, smoking, systolic BP, diastolic BP, HA1C, hemoglobin, serum uric acid, triglycerides, HDL-cholesterol, and albuminuria.

## Data Availability

The excel of individual clinical data used to support the findings of this study are available from the corresponding author upon request.
